# Vector Design for Improved DNA Vaccine Efficacy, Safety and Production

**DOI:** 10.3390/vaccines1030225

**Published:** 2013-06-25

**Authors:** James A. Williams

**Affiliations:** Nature Technology Corporation/Suite 103, 4701 Innovation Drive, Lincoln, NE 68521, USA; E-Mail: jim@natx.com; Tel.: +1-402-323-6289; Fax: +1-402-323-6292

**Keywords:** DNA vaccination, plasmid, antibiotic-free, non-viral, fermentation, immunization, adjuvant, innate immunity

## Abstract

DNA vaccination is a disruptive technology that offers the promise of a new rapidly deployed vaccination platform to treat human and animal disease with gene-based materials. Innovations such as electroporation, needle free jet delivery and lipid-based carriers increase transgene expression and immunogenicity through more effective gene delivery. This review summarizes complementary vector design innovations that, when combined with leading delivery platforms, further enhance DNA vaccine performance. These next generation vectors also address potential safety issues such as antibiotic selection, and increase plasmid manufacturing quality and yield in exemplary fermentation production processes. Application of optimized constructs in combination with improved delivery platforms tangibly improves the prospect of successful application of DNA vaccination as prophylactic vaccines for diverse human infectious disease targets or as therapeutic vaccines for cancer and allergy.

## 1. Introduction

DNA vaccines are plasmids that combine sequences required for replication and selection in *Escherichia coli* (bacterial region) with sequences necessary to express an encoded transgene in vertebrate cells (eukaryotic region) after delivery to an organism and transfection of target tissue cells. A great deal has been learned about the mechanism of action of DNA vaccination since the first publication in 1992. After delivery to the patient, the vector encoded transgene antigen is transcribed after entering the cell nucleus. The mRNA is exported to the cytoplasm and subsequently translated. The host-expressed antigen is presented to the immune system by either major histocompatibility complex (MHC) class I or II. Transfected DNA also activates innate immunity which is critical to promote an immune response against the MHC presented antigen [1,2,3]. DNA vaccines are inherently safe since the vectors are non-replicating, encode and express only the target antigen, and are not live and therefore cannot revert to a disease causing form as with viral vectors. A key advantage of DNA vaccination is that, unlike viral vector particles, DNA vaccines do not induce anti-vector immunity, and therefore may be utilized in prime and boost regiments and with multiple products intended for the same patient. DNA vaccination is also effective in neonates even in the presence of maternal neutralizing antibodies [4]. Additionally, DNA vaccine manufacture is much easier and faster than alternative vaccine platforms and the DNA product is highly stable. DNA vaccines are well tolerated and have an excellent safety profile in human clinical investigations with no reported safety concerns such as DNA integration into the host genome, antigen tolerance or autoimmunity (reviewed in [1,2,3]). 

The licensure of four animal health DNA vaccine products demonstrates the utility of DNA vaccination in large animals including horses and pigs. These licensed products include preventative vaccines for West Nile virus in horses and infectious haematopoietic necrosis virus in fish, a therapeutic cancer vaccine for dogs, and a growth hormone gene therapy to increase litter survival in breeding pig sows [1]. DNA prime-heterologous boost vaccination with influenza hemagglutinin antigen has demonstrated utility to induce broadly cross neutralizing antibodies [5,6,7]. However, efficient plasmid delivery is often required to generate protective responses in large animals and humans compared to mice. Various DNA delivery platforms have been developed that have demonstrated promising results in large animals and humans, including electroporation (EP) [8], needle free jet-injection [9,10] and lipid [11] deliveries. Interestingly, human serum amyloid P binds and inhibits plasmid transfection and DNA vaccine induced adaptive immune responses much more strongly than the murine counterpart [12,13]. This and other species specific differences may collectively account for the greater difficulty in obtaining acceptable efficacy in humans.

Most delivery platforms such as electroporation greatly increase plasmid transfer across the cell plasma membrane barrier to directly or indirectly transfect plasmid into the cell cytoplasm but do not deliver DNA to the nucleus [14]. Plasmid transfection into the cell, and vector diffusion through the cytoplasm and nuclear uptake may be enhanced using smaller more compact vectors or nuclear targeting sequences [15]. Within the nucleus, transgene expression levels may be dramatically increased by optimization of the bacterial and eukaryotic regions. In this review, vector innovations that improve DNA vaccine performance are discussed. Critical issues for plasmid manufacturing are also discussed, and exemplary plasmid production processes highlighted.

## 2. Plasmid Design

DNA vaccine vectors combine a eukaryotic region that directs expression of the transgene in the target organism with a bacterial region that provides selection and propagation in the *Escherichia coli* (*E. coli*) host. The eukaryotic region contains a promoter upstream, and a polyadenylation signal (polyA) downstream, of the gene of interest. Upon transfection into the cell nucleus, the promoter directs transcription of an mRNA that includes the transgene. The polyadenylation signal mediates mRNA cleavage and polyadenylation, which leads to efficient mRNA export to the cytoplasm. A Kozak sequence (gccgccRccATGG consensus, transgene ATG start codon within the Kozak sequence is underlined, critical residues in caps, R = A or G) is included. The Kozak sequence is recognized in the cytoplasm by ribosomes and directs efficient transgene translation. The constitutive human Cytomegalovirus (CMV) promoter is the most common promoter used in DNA vaccines since it is highly active in most mammalian cells transcribing higher levels of mRNA than alternative viral or cellular promoters. PolyA signals derived from the rabbit β-globin or bovine growth hormone genes are typically used. These signals contain accessory sequences upstream and downstream of the polyadenylation site (AATAAA) that increase polyadenylation efficiency resulting in increased mRNA levels, and improved transgene expression. 

**Figure 1:**
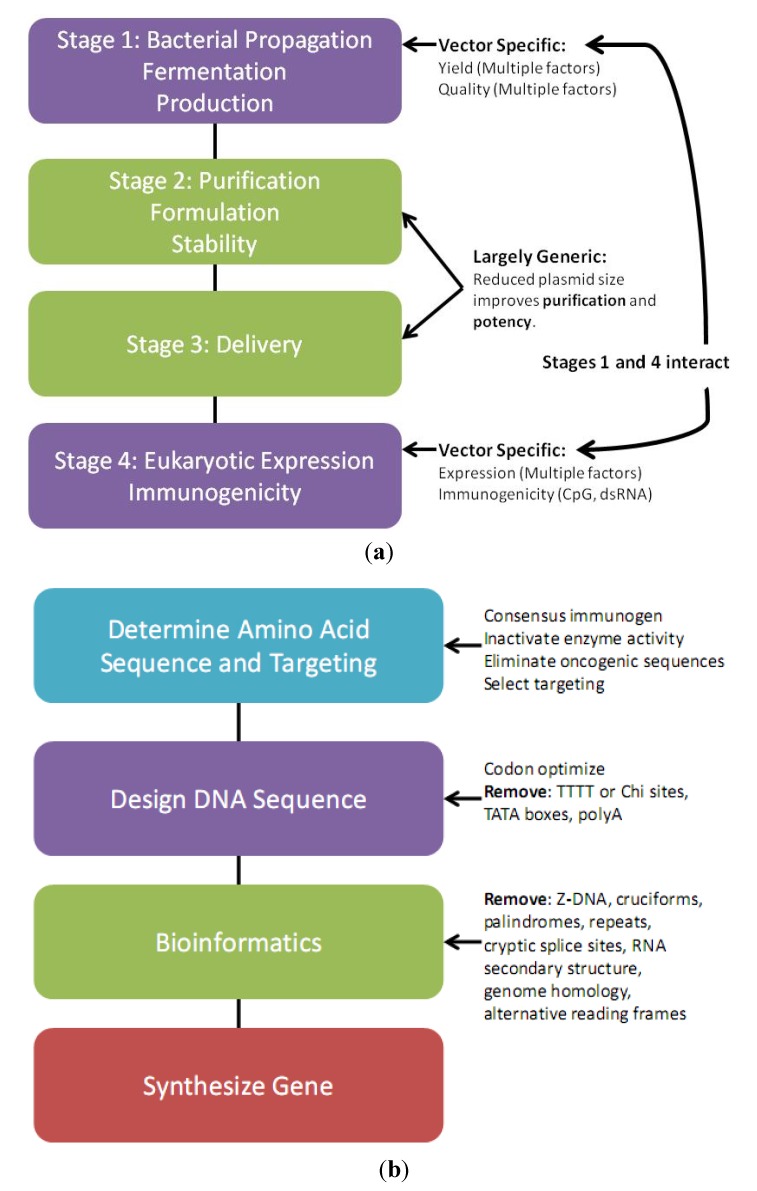
(**a**) DNA vaccine vector production and application flowchart. Stages 1 and 4 are very sensitive to vector changes and must be optimized coordinately since vector modification to enhance one parameter can have multiple undesired effects on other parameters. Stages 2 and 3 are largely generic; (**b**) Insert design flowchart.

The transcribed 3' and 5' untranslated regions (UTRs) flanking the transgene should not contain open reading frames (ORFs) since ORFs in these regions have been shown to be translated into immunogenic peptides [16]. The bacterial region combines a high copy replication origin, most usually the pUC origin, with a selectable marker. Perhaps surprisingly, certain orientations and compositions of bacterial region sequences can dramatically reduce eukaryotic region directed transgene expression, manufacturing yields, and plasmid quality in the *E. coli* host [17,18]. Reduced expression with certain backbones may in part be due to production, from cryptic promoters in the vector backbone, of double stranded RNA (dsRNA) that triggers protein kinase R (PKR) mediated translational shutdown or RNA interference [19,20]. Thus, since both bacterial production and expression in the target organism are very sensitive to vector changes (Figure 1a), a critical part of vector design is careful selection and assembly of bacterial region selection and replication sequences.

**Figure 2:**
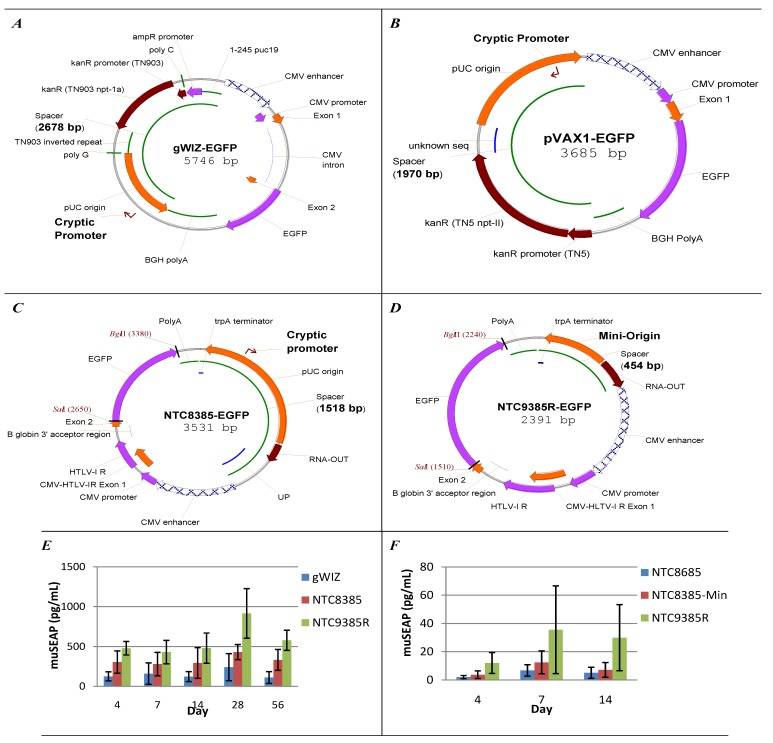
DNA vaccine vectors. (**a**,**b**) 1st; (**c**) 2nd; and (**d**) 3rd; generation DNA Vaccine vectors; (**e**) 2nd and 3rd generation vectors increase *in vivo* expression compared to first generation vector gWIZ. 5 µg muSEAP vectors delivered intramuscularly with EP to mice on day 0, serum muSEAP assayed on indicated days. 3rd generation vector NTC9385R has significantly higher expression than gWIZ or 2nd generation vector NTC8385 (*p*-value = 0.05; Mann-Whitney rank-sum test); (**f**) 3rd generation vectors dramatically increase *in vivo* expression, compared to 2nd generation. 50 µg muSEAP vectors in 50 µL saline delivered intradermally to mice with EP on day 0, muSEAP assayed on indicated days. 3rd generation vector NTC9385R has significantly higher expression than 2nd generation vector NTC8685 (*p*-value = 0.05; Mann-Whitney rank-sum test). NTC8685 is a 2nd generation vector similar to NTC8385. The NTC8385 1,518 basepair (bp) bacterial region (spacer region) is reduced to 855 bp in NTC8385-min and 454 bp in NTC9385R. This compares to 2,678 bp for gWIZ, and 1,970 bp for pVAX1.

First generation DNA vaccine vectors such as pVAX1 (Invitrogen; Figure 2b) and gWIZ (Genlantis, Figure 2a) contain the kanamycin resistance (kanR) gene as a selectable marker. pVAX1 is a basic vector that contains no eukaryotic or bacterial region optimizations, and consequently has relatively low manufacturing yield and expression *in vitro* and *in vivo* (mice) [21]. pVAX1 expression is reduced, compared to alternative CMV promoter vectors, by inhibitory sequences in the bacterial region (see Section 5.1). The pUC origin is oriented such that the pUC origin encoded cryptic eukaryotic promoter [19] will transcribe RNA antisense to the transgene (Figure 2b); this may produce dsRNA and reduce expression by RNA interference or PKR mediated translational inhibition. The gWIZ vector has 5-fold improved expression and 2-fold increased manufacturing yields relative to pVAX1 [21] due to extensive optimization of the orientation and composition of the bacterial region [17] and addition of an intron upstream of the transgene.

### 2.1. Vector Design Considerations

New vectors have been constructed that combine improved transgene expression with superior manufacturing yields and regulatory compliance compared to first generation vectors. Design criteria are outlined below and example vectors described in Section 2.2. See [22,23,24] for comprehensive reviews on DNA vaccine vector and insert design.

Regulatory: The FDA and European Union (EU) have issued guidance documents that include vector design considerations for plasmid DNA vectors intended for human use [25,26,27]. Vectors should be minimalized to remove extra nonfunctional sequences, especially ones that encode cryptic ORFs that may be expressed in the target organism. This is especially critical within the transcribed UTRs to prevent production of vector encoded cryptic peptides in the target organism [28] that may induce inappropriate adaptive immune responses [16,29]. For example, the mRNA nuclear export enhancing Hepatitis virus derived posttranslational regulatory element (PRE) included within the transcription unit downstream of the stop codon in some vectors encodes a 178 bp amino acid fragment of the viral polymerase gene [22]; immune response against this viral protein may alter immune responses in individuals with prior exposure and circulating T-cells to Hepatitis. In addition to general concerns regarding use of antibiotic selection markers, the European Union has specifically recommended the elimination of kanR selection markers [27] (see Section 2.2). Vector retrofit to replace the kanR marker with short RNA antibiotic-free markers has the unexpected benefit of improved expression (see Section 5.1).

Expression: The 5' UTR upstream of the transgene is typically 50–150 bp and contains a Kozak sequence with no additional ATG motifs upstream of the Kozak sequence that could function as unintended start codons. As well, stable mRNA secondary structures that include the authentic ATG containing Kozak sequence are eliminated since they may reduce translation by prevention of Kozak sequence mediated ribosome recruitment. An intron within the eukaryotic region 5' UTR improves transgene expression [2]. Intron splicing and ultimately transgene expression may be further improved by intron optimization through the addition of splicing enhancers within and flanking the intron [30,31,32]. Insertion of the human T-cell leukemia virus type I R region (HTLV-I R) 5' UTR downstream of the CMV promoter enhances mRNA translation efficiency and further increases transgene expression in mice and nonhuman primates [28,33]. HTLV-I R encoding DNA vaccines have an excellent safety profile established in multiple human clinical trials [7]. To prevent transgene-directed dsRNA formation that may result in RNA interference mediated transgene silencing, bacterial region sequences should not contain cryptic eukaryotic promoters oriented antisense to the transgene [20]. Comparing expression between different constructs in mammalian cells to select an optimal vector must be done carefully since transgene mRNA levels can easily saturate protein production capacity *in vitro* [34]. Interestingly, minimalization of the bacterial region has recently been demonstrated to improve transgene expression (see Section 5.2). 

Manufacture/quality: First generation vectors were not optimized for production yield and quality, which can impose significant cost post-licensure. Ideally a plasmid is predominantly monomer with a low propensity for nicking or rearrangement during fermentation, or nicking or denaturation during extraction and downstream purification. Unusual DNA sequences such as runs of homopurine-homopyrimidine tracts, inverted or direct repeats may be prone to instability. Palindromes are unstable and reduce plasmid copy number. AT-rich sequences and cruciforms increase the frequency of plasmid nicking, while Chi sites mediate plasmid multimerization [22,23,35]. Cryptic bacterial promoters within the eukaryotic promoter region may lead to inappropriate expression of the transgene in the bacterial host. In many cases this will be toxic [22], reducing plasmid stability and production yields and will require the creation of designer strains that express transgene-complementary RNA from the bacterial chromosome to prevent translation of the toxic protein [36]. The presence of such undesired sequences in a vector (or gene insert, see Section 2.3) may be identified using bioinformatics [22,37]. 

### 2.2. Antibiotic-Free Selection Using RNA Selection Markers

The use of antibiotic resistance markers in DNA vaccines has potential regulatory safety concerns. These include production mediated environmental contamination with either antibiotics used in fermentation culture or the plasmid borne antibiotic resistance markers [38], treatment associated transfer of antibiotic resistance to a patient’s endogenous microbial flora (e.g., transfection of skin resident microorganisms with topically applied plasmid DNA), or activation and transcription of the marker from host cell promoters after spurious incorporation into the cellular genome after transfection of the patient’s cells. Selection using ampicillin during production is generally not acceptable due to potential hyper reactivity to residual trace β lactam antibiotics in the product. The European Pharmacopoeia indicates that “Unless otherwise justified and authorised, antibiotic-resistance genes used as selectable genetic markers, particularly for clinically useful antibiotics, are not included in the vector construct. Other selection techniques for the recombinant plasmid are preferred” [39]. The European Medicines Agency (EMA) has further concluded that kanamycin and neomycin are of importance for veterinary and human use and cannot be classified as having minor therapeutic relevance due to current use in critical clinical settings [27]. To address these regulatory concerns, alternative non-antibiotic selection methods are needed. 

**Figure 3:**
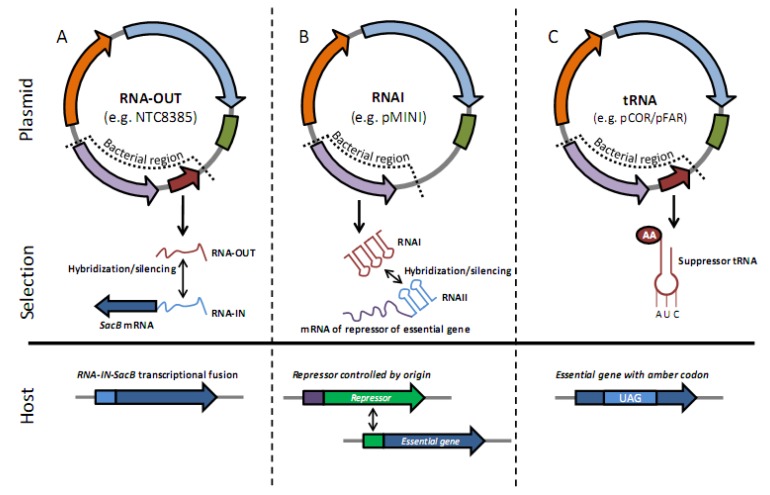
RNA selectable marker DNA vaccine plasmids. Purple arrow in bacterial region is pUC replication origin, brown arrow in panels (**a**) and (**c**) is the RNA selection marker. Eukaryotic region promoter, transgene and polyA are depicted with orange arrow, blue arrow and green box, respectively. (**a**) NTC8385 plasmid borne RNA-OUT RNA binds a chromosomally encoded constitutively expressed mRNA that contains the RNA-IN target sequence in the leader. This prevents translation of the downstream levansucrase (sacB), allowing growth on sucrose media; (**b**) pMINI pUC origin encoded RNAI binds a chromosomally encoded constitutively expressed mRNA that contains the RNAII target sequence in the leader. In the *murselect*-system, an essential gene (*murA*) is modified to contain a repressor binding site in the promoter and the RNAII target sequence is incorporated into the repressor mRNA leader. RNAI binding to RNAII prevents repressor translation, allowing expression of the essential gene; (**c**) pFAR4/pCOR plasmid borne suppressor tRNA allows read-through translation of an amber nonsense codon in a chromosome encoded essential gene. Adapted from Oliveira and Mairhofer, 2013 [48].

The use of any protein-based selection marker raises the concern that it may be unintentionally expressed and translated in the vaccinated organism. While a number of antibiotic-free (AF) plasmid retention systems have been developed in which the vector-encoded selection marker is not protein based [22,40] superior expression and manufacture has been observed with DNA vaccine vectors that incorporate RNA based antibiotic-free selection markers. For example, the NTC8385 sucrose selection vector (Figure 2c) encodes RNA-OUT, a small 70 bp antisense RNA (Figure 3a) [41]; pFAR4 and pCOR vectors encode a nonsense suppressor tRNA marker (Figure 3c) [42,43], while the pMINI vector utilizes the ColE1 origin-encoded RNAI antisense RNA (Figure 3b) [44,45]. These plasmid borne RNAs regulate the translation of a host chromosome encoded selectable marker allowing plasmid selection (Figure 3). Of these, high yield fermentation processes (>500 mg/L) have been developed for RNA-OUT vectors (1,800 mg/L; [46]) and pMINI (900 mg/L; [47]). In all these vectors, replacement of the kanR antibiotic selection marker resulted in increased transgene expression in the target organism (see Section 5) demonstrating elimination of antibiotic selection to meet regulatory criteria may unexpectedly also improve product performance (reviewed in [48]). 

### 2.3. Transgene Design Considerations

DNA vaccines, due to *in vivo* antigen expression, have the advantage that vaccinologists may easily customize encoded antigens through rational transgene design. Commercial gene synthesis has become rapid and inexpensive, thus enabling DNA vaccine antigen design, synthetic codon optimized antigen gene synthesis and vaccine manufacture on a highly compressed timeline. 

Antigen transgenes for inclusion in DNA vaccines may be an exact copy of the original antigen or a modification to improve efficacy or safety. Antigens may be altered to inactivate enzymatic activity, remove potentially oncogenic sequences or attenuate virulence. Mutations to reduce DNA binding may mitigate concern that immune responses against DNA will be induced by protein/nucleic acid complexes. Alternatively, the antigen may be extensively engineered for immunogenicity using structure based antigen design [49]. 

For pathogens that contain multiple serotypes, rather than using multiple plasmids, engineering a single broadly cross neutralizing antigen is a possibility. Two technologies that may be used to accomplish this are a bioinformatics approach that generates a consensus immunogen [50] or a directed molecular evolution approach that uses molecular breeding to evolve genes through an iterative process consisting of recombinant generation *in vitro* followed by selection of cross neutralizing recombinants [51]. 

Adaptive immune responses may be improved by enhancing antigen processing and MHC class I and/or class II presentation [1,2]. This can be accomplished by the addition of a targeting peptide that routes antigens to various intracellular destinations. DNA vaccine antigens are most commonly targeted to the secretion pathway using a signal peptide [52]. This may use a heterologous secretion signal, or, in the case of a secreted protein, the native secretion signal. Use of an optimized signal sequence may dramatically improve expression over the native sequence. Improvement has been observed using an optimized tissue plasminogen activator (TPA) signal peptide [52,53,54] or IgE gene leader [2]. An optimized heterologous secretion tag is often included in DNA vaccine vectors and the transgene is cloned downstream and in frame with the signal peptide [18]. Alternatively the signal peptide may be included when designing the synthetic gene. 

In some DNA vaccines, proteosomal targeting using an *N*-terminal ubiquitin tag (terminal ubiquitin G76 residue altered to A76 to destabilize the fusion protein) is used to promote MHC class I antigen presentation [55] while endosomal targeting by transgene insertion within the LAMP protein is used to promote MHC class II antigen presentation [56]. To experimentally determine optimal antigen targeting, a family of antigen targeting, RNA selectable marker (RNA-OUT), optimized DNA vaccine plasmids with compatible cloning into vectors encoding either *N*-terminal TPA signal peptide (secretion targeting), *N*-terminal and *C*-terminal LAMP1 (endosomal targeting) or *N*-terminal destabilizing Ubiquitin A76 (proteosome targeting) are commercially available (Nature Technology Corporation, Lincoln, NE, USA). 

Many other targeting tags have also been described [1,22] including transgene fusion N-terminal to strong immunogens that contain MHC class I and/or MHC class II binding peptides. Fusion to MHC class II peptides that induce CD4+ T-cell help may improve antibody or cytotoxic CD8+ T cell responses [57].

Once the antigen protein sequence is finalized, a synthetic gene sequence is designed (Figure 1b), synthesized and cloned into a vector downstream of a consensus Kozak sequence to ensure efficient translation. The optimized protein sequence is reverse translated into a gene sequence, selecting optimal codon usage for the target species. Codon optimization to match high use codons for the target species has been shown to dramatically increase transgene expression [53,58]. Elimination of extensive RNA secondary structure is also important. Some codon optimization programs such as the GeneArt GeneOptimizer^®^ Process combine RNA and codon optimization [58]. An important consideration is that RNA secondary structure between the synthetic gene and the vector 5' UTR is not screened by gene synthesis companies. Secondary structure between the synthetic gene and the 5' UTR encoded Kozak sequence may interfere with ribosome recruitment and reduce transgene expression. Such hybrids may be detected using a program such as mfold [59]. The gene sequence is synthesized and cloned into the DNA vaccine vector backbone.

Synthetic gene design is critical. FDA guidance indicates “biodistribution studies may be waived for DNA vaccines produced by inserting a novel gene into a plasmid vector previously documented to have an acceptable biodistribution/integration profile” [25]. Thus a new gene to be inserted into a previously validated vector must not create regulatory concerns due to trivial design issues. New synthetic genes should be screened using the same criteria as described above for vector design to eliminate unusual DNA sequences (e.g., G quadruplex), inverted or direct repeats, palindromes, AT-rich sequences and cruciforms, Chi sites and cryptic bacterial promoters that could affect plasmid quality. Additionally, it is critical to ensure no cryptic splice acceptor or donor sites (sense orientation), polyadenylation sites (AATAAA or ATTAAA), or eukaryotic promoters (both orientations) are present within the insert, since this could result in the generation of aberrant peptides causing regulatory concern [2,22]. Complementary strand promoters would transcribe mRNA that would anneal to the transgene mRNA to create dsRNA that may silence transgene expression. Additionally, as a precaution to reduce potential regulatory agency concern regarding the theoretical risk of insertional mutagenesis of the host genome, large tracks of sequence homology to the target organism identified using the National Center for Biotechnology Information (NCBI) BLASTN program should be removed. A codon optimized synthetic gene typically contains regions with only short tract homology of less than 20 bps of perfect identity to a target genome. These short tracts of homology should not be an issue since characterization of plasmid DNA integration into the genome using repeat-anchored integration capture (RAIC) PCR has demonstrated short homology driven integration events are extremely rare [60]. See Figure 1b and [22] for a detailed insert design flowchart. 

## 3. Plasmid Manufacture

Several critical factors should be considered prior to large scale cGMP manufacturing for clinical investigations, including product purity and homogeneity (*i.e*., percent covalently closed monomeric plasmid DNA) specifications, product concentration and formulation, and projected quantities needed for clinical trials and commercialization. Unfortunately, most first generation DNA vaccine vectors are not optimized for fermentation yield and homogeneity and are nicking or dimerization prone [22]. Poor quality is a critical problem, since vector redesign and sequence modification to improve quality to meet clinical specifications may necessitate additional expensive non-clinical toxicology testing to be performed which would delay clinical evaluation. Poor production yield is problematic down the road since it will impose significant cost burden post-licensure. 

### 3.1. Plasmid Fermentation

In general, plasmid quality and yield is higher from fed-batch rather than batch fermentation. A few high yield fed-batch plasmid fermentation processes (500–2,600 mg/L) have been described. These processes all couple reduced growth rate (which generally increases copy number) with high copy replication origins [61]. One of these, the patented HyperGRO^TM^ inducible fed-batch fermentation process [62], has been utilized to manufacture clinical grade DNA for various plasmids and is generally available for commercial production of research grade (Nature Technology Corporation, Lincoln, NE, USA) or clinical grade plasmid DNA through licenses to several cGMP plasmid manufacturers, including Aldevron, Eurogentec and VGXI. HyperGRO^TM^ incorporates novel cell bank and fermentation process innovations that reduce plasmid mediated metabolic burden allowing generic production of a wide range of plasmids with low levels of dimerization or nicking and high fermentation productivity up to 2,600 mg/L [63]. High plasmid homogeneity in the fermentation harvest is critical, since removal of nicked plasmid and dimers is extremely difficult due to similar properties to the desired supercoiled plasmid monomer product. Likewise, high yield is important since increased plasmid yield per gram of bacteria results in improved final product purity [61]. An alternative commercially available high yield fermentation process has been developed by Boehringer Ingelheim and is available for clinical production of plasmid DNA vaccines at their facilities [64].

### 3.2. Downstream Plasmid Purification

Following fermentation, plasmid DNA is typically extracted using alkaline lysis. Alkaline lysis is difficult to scale, but a number of companies have developed mixing methodologies that remove host cell DNA fragments without denaturing or nicking plasmid DNA. Most commercial manufacturers have developed downstream purification processes that maintain plasmid quality while removing impurities such as endotoxin, genomic DNA, bacterial RNA, and nonsupercoiled plasmid isoforms, for example, anion exchange chromatography followed by hydrophobic interaction chromatography. The reader is directed to several detailed reviews of downstream plasmid purification [61,65,66,67,68]. 

Combining a fermentation process such as HyperGRO^TM^, that generates high quality supercoiled monomer plasmid with low dimerization and nicking, with an alkaline lysis extraction-downstream purification process optimized to not denature or nick plasmid DNA will provide a plasmid product that will meet stringent plasmid homogeneity specifications [46]. 

### 3.3. Plasmid Quality Control Considerations

The downstream purification process must remove impurities such as protein, RNA, chromosomal DNA, and endotoxins to acceptable levels. Of these, chromosomal DNA is the most difficult to remove due to similar properties to the plasmid product; thus optimization of alkaline lysis to prevent chromosomal DNA extraction is critical since poor alkaline lysis can result in elevated levels of chromosomal DNA in the final product. While impurity levels for clinical investigation may be relatively easy to achieve (typically <1% protein, RNA, chromosomal DNA impurities, <10 endotoxin units/mg plasmid [61]) the final specifications for protein and chromosomal DNA for commercial use may be tighter since licensed protein products have much lower residual host protein (typically <100 ppm) and gDNA (typically <100 pg/dose) limits [61]. The final commercial specifications may depend on dose, delivery and regulatory agency input. 

*E. coli* derived impurities may also detrimentally affect vaccine performance. For example, genomic DNA has been shown to cause skeletal muscle damage after hydrodynamic limb vein delivery [69] and inflammation after lipoplex gene delivery to the lung [70]. Colanic acid polysaccharide impurities in plasmid DNA cause acute toxicity after intravenous injection of plasmid liposome complexes [71]. Critically, impurities such as genomic DNA, ribosomal RNA or endotoxin are ligands of various innate immune receptors. Thus the presence of these impurities may activate innate immunity, inflammation responses, and alter adaptive immunogenicity in a lot to lot, or species-specific fashion. For example, the orphan murine receptor TLR13 triggers cytokine secretion in response to bacterial ribosomal RNA [72]). 

Potency assays (*in vitro* and/or *in vivo*) are product specific and are designed to measure the biological activity of each DNA vaccine lot *versus* a reference standard to ensure lot to lot vaccination consistency. Typically for early clinical development, an *in vitro* assay measuring transgene expression after transfection is proposed as a surrogate for immunogenicity [73,74]. However, evidence to support correlation of *in vitro* expression with *in vivo* immunogenicity may be required.

### 3.4. Plasmid Host Strain and Growth Conditions Affect DNA Vaccine Performance

Plasmid DNA production is typically performed in *endA* (DNA-specific endonuclease I), *recA* (DNA recombination) deficient *E. coli* K12 strains such as DH5α, DH5, DH1, XL1Blue, GT115, JM108, DH10B, or *endA*, *recA* engineered derivatives of alternative strains such as MG1655 [75] or BL21 [22,63,76]. 

Replication of pUC origin plasmids is dependent entirely on multiple *E. coli* host strain encoded factors [77]. Host encoded replication protein expression level variations between strains likely accounts for observable differences in plasmid properties such as percent open circular plasmid, steady state supercoiling density, catenation, multimerization and yield [78]. For example, high levels of open circle plasmid may be indicative of incomplete replication since plasmids retaining the RNA primer are nicked during alkaline lysis. Variations in open circular plasmid levels between strains may reflect altered levels of DNA Pol I and DNA ligase, since these enzymes are required to remove the replication initiating RNA primer, and create a covalently closed circular (CCC) plasmid, respectively [77]. Different plasmid isoforms may have altered transfection efficiency, intracellular stability, nuclear transfer rate, or promoter activity [22,63] that may dramatically affect transgene expression *in vivo* so it is critical to control DNA vaccine plasmid production conditions to ensure consistent product quality and *in vivo* performance during preclinical and clinical development.

Significantly, varying production conditions may affect host replication protein expression levels and/or activity which may alter plasmid properties [63]. Negative supercoiling, the under-winding of a DNA strand, is an epigenetic modification that may affect plasmid manufacture and transgene expression. The actions of DNA gyrase (gyrA, gyrB), which increases negative supercoiling, along with relaxing enzymes Topoisomerase I (topA) and Topoisomerase IV (parC, parE; also essential for unknotting plasmid catenates [79]) sets the steady state supercoiling density (σ) [80,81]. σ varies between strains and growth conditions such as growth temperature [82], growth phase [83] and can be perturbed by environmental stress such as nutrient limitation [84] or high temperature spikes during production [85]. Rapidly replicating pUC plasmids in fermentation cultures may not complete replication or reach steady state σ; introduction of a post plasmid production hold step at low temperature to reduce pUC plasmid replication initiation allows completion of initiated replication cycles and plasmid supercoiling to physiological levels [77]. 

Altering σ may alter transgene expression due to changed susceptibility to stress-induced duplex destabilization (SIDD). SIDD sites are found within transcriptional regulatory regions such as promoters [86] and origins of replication. The activity of many promoters is affected by σ alterations that change the susceptibility to SIDD [87]. Different steady state σ levels may also alter plasmid manufacturing yields between strains. Small plasmids less than 3 kb in size often have poor fermentation yields in standard strains such as DH5α, but can be produced to high yield in XL1Blue [21]: the increased negative supercoiling in XL1Blue relative to DH5α may alter the pUC origin susceptibility to SIDD, facilitating replication of small plasmids [21]. It is critical therefore that the plasmid supercoiling density be maintained during fermentation scaleup and clinical development by tight manufacturing control. 

Altering epigenetic DNA methylation may also affect transgene expression [63]. While all standard plasmid production strains encode epigenetic dam nucleotide methylation at GATC residues, plasmid from different strains may differ in: (1) strain-specific epigenetic dcm nucleotide methylation (at CCWGG; BL21 and GT115 are dcm-) and (2) negative supercoiling density as described above. From a regulatory perspective, a plasmid with modified epigenetic methylation is a distinct chemical entity and therefore a different product. It is critical therefore that the optimal strain/methylation for plasmid manufacture and performance be identified prior to product definition and subsequent clinical development. 

In summary, methylation and supercoiling should be monitored during production scaleup since plasmids may have altered biological properties (potency) due to incomplete methylation [63] or nonphysiological supercoiling [21]. Incomplete dam or dcm methylation may be detected by restriction endonuclease digestion (dam: Sau3A cleaves all sites, MboI cleaves unmethylated sites, DpnI cleaves methylated sites; dcm: BstNI cleaves all sites, EcoRII cleaves unmethylated sites) [63] and supercoiling linking number by chloroquine agarose gel electrophoresis [21].

Different strains have host chromosome encoded transposons that under stress conditions may transpose into plasmids, for example, IS1 into the neomycin resistance marker promoter during strain adaptation to defined media [88]. This generates a heterogeneous product of plasmid with and without insertion elements which is unacceptable for clinical use [89]. The HyperGRO^TM^ cell banking and fermentation process is designed to reduce metabolic stress, and has been shown to not induce IS1 transposon mobilization during cell banking or fermentation unit operations [63]. 

## 4. DNA Vaccine Immunology

Extensive research over the last two decades has identified intracellular DNA sensing pathways and mechanisms by which DNA vaccines activate these pathways to induce adaptive immunity (reviewed in [90,91,92]). The application of this knowledge to create strategies to improve DNA vaccine immunogenicity is discussed below.

### 4.1. DNA Vaccination Activates Innate Immunity

**Figure 4:**
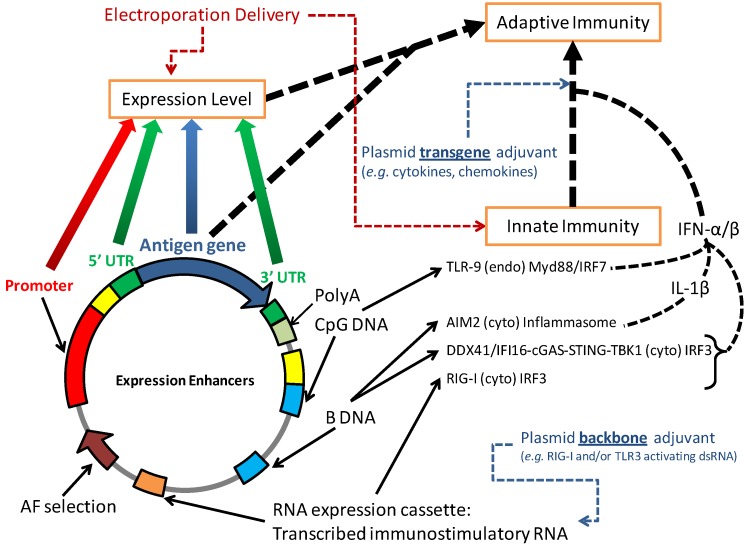
Molecular mechanisms of DNA vaccines. Transfected B DNA (the most common double helical DNA structure) is sensed in the cytoplasm (cyto) by DNA receptors interferon-inducible protein 16 (IFI16) and DEAD (Asp-Glu-Ala-Asp) box polypeptide 41 (DDX41) activating the cGAMP synthase (cGAS) [98] /STING/TBK1 pathway to induce type 1 interferon production and NF-κB. An additional cytoplasmic innate immune pathway activated by transfected DNA is the cytoplasmic AIM2 inflammasome. IFI16, DDX41 and AIM2 detect DNA generically and are not sequence specific although IFI16 may preferentially recognize DNA that forms cruciforms or is negatively supercoiled [99]. By contrast, specific CpG motifs in DNA vaccines are sensed by the endosomal (endo) TLR9 innate immune receptor. To improve innate immune activation, addition of optimized immunostimulatory CpG motifs in the vector backbone may be used to increase TLR9 activation while immunostimulatory RNA expressed from the vector may be utilized to activate alterative RNA sensing innate immune receptors such as RIG-I (plasmid backbone adjuvant). Due to limited transgene expression after DNA vaccination in large animals, vector modifications and deliveries that improve transgene expression also improve adaptive immunity. Certain delivery modalities such as EP that improve gene transfer efficiency also activate innate immunity through tissue damage [100,101,102]. EP conditions need to be carefully optimized, since the optimal EP conditions for DNA vaccination are not necessarily those with the highest gene expression [103] and optimal delivery parameters vary between strains [100].

Studies using knock-out mice deficient in various innate immune receptors and signaling molecules have determined that most of the “adjuvant effect” of DNA vaccination is mediated by activation of the cytoplasmic double stranded DNA sensing stimulator of interferon genes/TANK-binding kinase 1 (STING/TBK1) dependent innate immune signaling pathway (Figure 4; reviewed in [93]). This is the primary pathway necessary to induce antigen specific B cells and CD4+ T-cells in response to DNA vaccination. However, several studies have demonstrated a role of endosomal sequence specific CpG DNA sensing Toll-like receptor 9 (TLR9) signaling in priming CD8+ T cell responses [94,95]. Cationic liposome delivered plasmid DNA clearly activates a CpG dependent inflammation response in the lung [96], so the contribution of TLR9 to DNA vaccination induced adaptive immunity may be tissue and delivery specific. Cytoplasmic DNA may also activate the absence in melanoma 2 (AIM2) inflammasome [97], but a role of inflammasome activation and the resultant caspase 1 mediated interleukin-1β production in DNA vaccine immunology has not been established.

### 4.2. Vector Modifications to Increase Innate Immunity

DNA vaccination efficacy may be improved by codelivery by a plasmid encoding adjuvant proteins (Figure 4). Numerous adjuvant plasmids have been developed, including those that express cytokines (e.g., interleukin-12), chemokines (e.g., RANTES), costimulatory molecules (e.g., CD40), or signaling molecules [e.g., interferon regulatory factor-3 (IRF3)] (reviewed in [93,104,105]). An alternative approach is to modify the vector backbone to encode DNA or RNA based adjuvants (plasmid backbone adjuvant; Figure 4). Such modifications avoid the autoimmunity concerns from expressing a human protein and do not limit boosting or multiple product development since the backbone encoded DNA or RNA adjuvant will not be the target of adaptive immunity. As well, this antigen expressing cell-targeted limited immunostimulation approach using backbone modified vectors is safer than nonspecific global stimulation by coadministering a large adjuvant dose such as TLR9 CpG agonist or Melanoma Differentiation-Associated protein 5 (MDA5)/TLR3 agonist poly I:C. 

Since the ligands for most characterized DNA sensing pathways are not sequence specific, research to improve DNA vaccine immunogenicity by adding DNA motifs has focused on addition of CpG TLR9 agonists. This is complicated by the fact that the flanking sequence determines if a CpG motif is immunostimulatory or immunosuppressive, and that optimal CpG agonists are species specific [106]. Results to date have been variable, but improved immune responses with DNA vaccines incorporating additional CpG motifs have been obtained [107,108]. Some of the variability in response is probably due to unintended alterations of transgene expression from the CpG motif modified vector backbone (see Section 2) as well as differences between delivery modalities in efficiency of endosomal trafficking of CpG motif containing DNA vaccines for TLR9 activation. 

An alternative approach is to engineer the vectors to coexpress immunostimulatory RNA (isRNA) with antigen. The isRNA is transcribed by either RNA Pol II (isRNA encoded either downstream of transgene in the 3' UTR or in a second transcription unit) or RNA Pol III (isRNA transcribed independently from transgene in the vector backbone). Both RNA Pol II and RNA Pol III expressed isRNA have been shown to improve DNA vaccination induced antigen-specific humoral and/or cellular response [109,110,111]. 

## 5. New Developments

Increasing DNA vaccine-mediated transgene expression improves immune response in large animals and humans [2]. Recently, as highlighted below, dramatically improved vector expression has been obtained by bacterial region minimalization.

### 5.1. Vector Bacterial Region Inhibits Plasmid Expression

A number of bacterial sequences have been shown to inhibit transgene expression in eukaryotic cells [22] (see Section 2). For example, the TN5 encoded kanamycin/neomycin resistance marker is a potent transcriptional silencer that decreases expression from linked eukaryotic promoters [112]. The pVAX1 vector (Figure 2b) has a 1,970 bp bacterial region (spacer between the eukaryotic region polyA and CMV promoter) including this TN5 kanR marker. Dramatically increased expression has been observed with antibiotic-free RNA selection marker pVAX1 derivative vectors, in which the kanR marker is replaced with RNA-OUT (pVAX1-AF, 1,195 bp spacer region) [21], amber suppressor t-RNA (pFAR4, 1,040 bp spacer region) [43] or removed (utilizing the pUC origin RNAII marker for selection; pMINI, 734 bp spacer region) [113] (see Section 2.2 and Figure 3). An alternative interpretation of the improved expression with RNA selection marker retrofitted pVAX1 vectors is that improved expression is due to the reduced vector size. All these vectors encoding transgene are >2,000 bp, which should not have significantly improved cytoplasmic mobility compared to larger plasmids [114]. However, smaller vectors are more effectively transfected into the cell, leading to higher transgene expression [115,116]. 

### 5.2. Minimal Backbone Vectors Dramatically Improve Plasmid Expression

Bacterial regions of approximately 1,000 bp or larger, mediate transgene silencing in certain tissues (e.g., liver), while minicircle vectors, containing shorter spacers ≤500 bp, have sustained transgene expression [117]. Silencing may be mediated by the formation of inhibitory chromatin on nontranscribed spacer region sequences [118]. Transcription of a bacterial region in eukaryotic cells using a heterologous promoter improved transgene expression duration [119]. Short bacterial region DNA vaccine vectors may therefore have application to increase antigen expression duration. Persistent antigen expression may improve memory CD8+ T-cell maintenance [120,121]. Consistent with that, sustained expression minicircle vectors elicit superior CD8+ T cell responses compared to plasmid vectors [122]. Minicircle vectors are manufactured from plasmid vectors in the *E. coli* host via the action of phage recombinases on recognition sequences in the plasmid to create circularized bacterial and eukaryotic regions (minicircle) which are then separated. Minicircle vectors are not practical for DNA vaccine applications since production procedures are very inefficient with optimal reported yields of only 5 mg minicircle per liter culture [123].

The pMINI, pFAR4 and NTC8385 RNA selection marker vectors all utilize the pUC origin for selection which can be minimalized to 700 bp without compromising high copy number replication. Replacement of the pUC origin in NTC8385 (Figure 2c) with a minimalized pUC origin (NTC8385-min) reduced the origin-RNA-OUT bacterial region from 1,518 bp to 855 bp. Further reduction of bacterial region size to 454 bp was obtained by replacing the pUC origin with a 300 bp mini-origin (NTC9385R; Figure 2d). The NTC9385R vector bacterial region is below the size limit that mediates transgene silencing in minicircle vectors [117]. Surprisingly, transgene expression level is also dramatically improved with this short spacer region vector (Figure 2e,f) [124]. 

Similarly improved transgene expression, 2 to 10 fold higher than conventional vectors, have also been observed with novel vectors that contain no spacer region, in which an RNA-OUT-replication origin bacterial region is encoded within an intron of the eukaryotic transcription unit [125,126]. While the mechanism to explain transgene expression enhancement with short spacer region vectors is not clear, these novel vectors have exciting application to improve DNA vaccine performance through improved expression level and duration.

## 6. Conclusions

DNA vaccines are a new generation biotechnology product that is beginning to enter the marketplace. While not critical in murine models, increased antigen expression correlates with improved immunogenicity in humans and large animals [2]. As reviewed herein, next generation vector designs have been developed that improve antigen expression, manufacturing yield and quality, and regulatory compliance. Application of these improved vectors and high yield manufacturing methodologies will be critical to ensure efficacy, safety and cost effective manufacture of future DNA vaccine products.
